# Characteristics, management and outcome of prehospital pediatric emergencies by a Dutch HEMS

**DOI:** 10.1007/s00068-020-01579-8

**Published:** 2021-02-04

**Authors:** Michelle Berdien Oude Alink, Xavier Roland Johnny Moors, Senned Karrar, Robert Jan Houmes, Dennis Den Hartog, Robert Jan Stolker

**Affiliations:** 1 Department of Anesthesiology, Erasmus University Medical Center-Sophia Children’s Hospital Rotterdam, Rotterdam, The Netherlands; 2 HEMS, Erasmus University Medical Center, Rotterdam, The Netherlands; 3 Intensive Care and Department of Pediatric Surgery, Erasmus University Medical Center-Sophia Children’s Hospital, Rotterdam, The Netherlands; 4 Department of Surgery-Traumatology, Erasmus University Medical Center, Rotterdam, The Netherlands; 5 Department of Pediatric Anesthesiology, Erasmus University Medical Center Rotterdam-Sophia Children’s Hospital, P.O. Box 2040, 3000 CA Rotterdam, The Netherlands

**Keywords:** HEMS, pediatric, trauma, resuscitation

## Abstract

**Background:**

In prehospital care, the Helicopter Emergency Medical Service (HEMS) can be dispatched for critically injured or ill children. However, little detail is known about dispatches for children, in terms of the incidence of prehospital interventions and overall mortality. The primary objective of this study is to provide an overview of pediatric patient characteristics and incidence of interventions.

**Methods:**

A retrospective chart review of all patients ≤ 17 years who received medical care by Rotterdam HEMS from 2012 until 2017 was carried out.

**Results:**

During the study period, 1905 pediatric patients were included. 59.1% of patients were male and mean age was 6.1 years with 53.2% of patients aged ≤ 3 years. 53.6% were traumatic patients and 49.7% were non-traumatic patients. 18.8% of patients were intubated. Surgical procedures were performed in 0.9%. Medication was administered in 58.1% of patients. Cardiopulmonary resuscitation (CPR) was necessary in 12.9% of patients, 19.9% were admitted to the intensive care unit and 14.0% needed mechanical ventilation. Overall mortality was 9.5%. Mortality in trauma patients was 5.5% and in non-trauma group 15.3%. 3.9% of patients died at the scene.

**Conclusions:**

Patients attended by HEMS are at high risk of prehospital interventions like CPR or intubation. EMS has little exposure to critically ill or injured children. Hence, HEMS expertise is required to perform critical procedures. Trauma patients had higher survival rates than non-traumatic patients. This may be explained by underlying illnesses in non-traumatic patients and CPR as reason for dispatch. Further research is needed to identify options for improving prehospital care in the non trauma pediatric patients.

## Introduction

The Dutch Emergency Medical Service (EMS) was dispatched 1.3 million times in 2016 for emergency calls and planned transport between hospitals. Of these patients 5.4% were < 16 years of age [1]. Similar rates of 5–10% pediatric patients were found in cohorts from Canadian EMS, United States EMS and Helicopter Emergency Medical Service (HEMS) and Austrian HEMS studies [2–6]. At this moment, there are little to no data on Dutch pediatric HEMS.

Dutch Helicopter Emergency Medical Service (HEMS) is staffed by an anesthesiologist or trauma surgeon and specialized nurse to provide advanced prehospital emergency care to patients of all ages and all types of injuries or illnesses. HEMS is dispatched in addition to EMS when advanced prehospital intervention is expected. This is assessed based on the information given during the initial emergency call and according to standard protocol [7].

In The Netherlands, the EMS crew works according to strict protocols describing the procedures they can perform, medications they can administer and in which circumstances [8]. Additional prehospital interventions by HEMS include rapid sequence induction (RSI), advanced or surgical airway management, chest tube placement, resuscitative thoracotomy, additional medication for pain relief and cardiovascular support with vasoactive medication.

The HEMS and EMS crews can perform several lifesaving interventions on scene. However, several studies show that EMS often do not have to perform these lifesaving interventions in pediatric patients. A Canadian EMS study showed a low rate of prehospital interventions in children by EMS such as intravenous medication (1.4%), bag-valve-mask (BVM) ventilation (0.3%) and intubation (0.1%) [2]. Similar rates were found in the United States and Belgium with critical procedures performed in 1–2% of pediatric calls [4, 6, 9, 10]. Austrian HEMS had higher, but still very low rates of advanced life support measures, with intubation in 3.7% and cardiopulmonary resuscitation (CPR) for 1.9% of the patients [3]. The majority of pediatric patients in the EMS and HEMS cohorts suffered (minor) trauma, respiratory distress or seizures [2–4, 6, 9–11].

To our best knowledge, there are little data about prehospital care for children and outcome. One EMS cohort showed a survival of 100%, whilst others showed a 4–8.4% mortality in HEMS and 4.8–6% in EMS trauma patients [2, 11–13]. Patients transported by HEMS demonstrate a better survival, probably due to the advanced prehospital interventions by the HEMS physician [14, 15]. However, the background of the medical teams differs between countries.

This study is aimed to obtain a better understanding of the nature of the full range of Dutch pediatric HEMS dispatches, required prehospital interventions and the outcome in terms of hospital stay and mortality.

## Ethics approval

The Medical Ethical Committee of the Erasmus University Medical Center Rotterdam (MEC-2017-351) approved this study.

## Methods

### Setting

The Netherlands has a population of 17.02 million. There are four physician staffed HEMS teams in the Netherlands that provide additional medical aid to the nurse-based EMS. The Rotterdam HEMS covers the South-West region of the country with the crowded urban province ‘Zuid-Holland’ with 3.7 million residents on 3403 km^2^ as well as the rural province ‘Zeeland’ with 380,000 residents on 2933 km^2^. The HEMS regions assist each other when there are several simultaneous dispatches in one region or there is a major disaster.

Dutch EMS crews consist of a specialized nurse; most have several years of experience in the intensive care unit, emergency room or anesthesia department. They receive an additional training of 9 months. All EMS nurses are trained in advanced life support and can perform several prehospital procedures according to national EMS protocols, such as endotracheal intubation of the adult patient during CPR and needle thoracocentesis in a tension pneumothorax [8].

The EMS nurse is assisted by an ambulance driver who is trained to assist the nurse during life support procedures. In situations where the EMS crew controls the situation using their standard national protocols and procedures, and where they do not expect any need for more advanced interventions they may decide to cancel the HEMS dispatch, according to protocol [7].

Dutch HEMS is physician based and uses a helicopter to provide quick access for the crew to patient. A rapid response ground vehicle is available for situations in which transport by ground is faster or when the weather conditions do not allow helicopter dispatch. If the patients require transport to a hospital, the first choice is transport by ground ambulance together with EMS crew, this is usually the fastest option in the Netherlands to reach a hospital. Another advantage of the EMS vehicle is that there is more space for the crew to provide care to the patient during transport. If the patient is located in a remote area, the site is not easily accessible, or a suitable hospital is too far away by ground the HEMS crew can transport the patient by air.

### Definition of criteria

We defined pediatric patients as being ≤ 17 years at the moment of dispatch. Trauma calls were defined as mechanically induced injuries such as traffic accidents, sports accidents, strangulation, burns and drowning. Non-trauma calls were defined as non-mechanical problems such as respiratory distress, seizures, CPR, anaphylaxis, new-born transition problems, a sick child and intoxication. If there was an overlap between reasons for dispatch, the patient was included in both groups.

### Data collection and analysis

All deployments of the Rotterdam physician-based HEMS were retrospectively reviewed from the HEMS database that registers all the dispatches. Patients that were ≤ 17 years of age at the moment of the dispatch were included in the 6-year period from January 2012–December 2017. HEMS dispatches that were canceled before evaluation of the patient by the HEMS crew were excluded.

Patient charts from Rotterdam HEMS were reviewed based on patient characteristics, such as age and gender, medical history, medication use, mechanism of injury or illness, prehospital diagnosis, prehospital intervention and mortality. In-hospital data were retrieved from the Erasmus University Medical Centre-Sophia Children’s Hospital if the patients was transferred to this hospital, data collected included emergency department diagnoses and treatment, hospital stay, ICU stay, interventions and surgical procedures.

For mortality rates, we used the available data of the initial HEMS assessment and for patients transported to the Erasmus University Medical Centre Rotterdam-Sophia Children’s Hospital we completed this with data fromthe electronic patient information systems.

For patients where death could not be confirmed through our primary sources, we requested information from the “Basis Registratie Personen” where all Dutch citizens are registered by name, gender, date of birth and if applicable the date of demise. These data were requested in April 2018. Follow-up of mortality was, therefore, a minimum of 3 months, and a maximum of 6 years and 3 months.

All data were analyzed using descriptive statistics with IBM SPPS Statistics version 24.0.0.1.

### Patient involvement

Patients and/or their legal guardian were not involved in the study design and conduction of this study.

## Results

### Inclusion and exclusion

During the research period of January 2012 until December 2017, there were 8,968 dispatches where Rotterdam HEMS arrived on the scene; 1905 (27%) of the dispatches were for a pediatric patient.

### Patient and dispatch characteristics

**Fig. 1:**
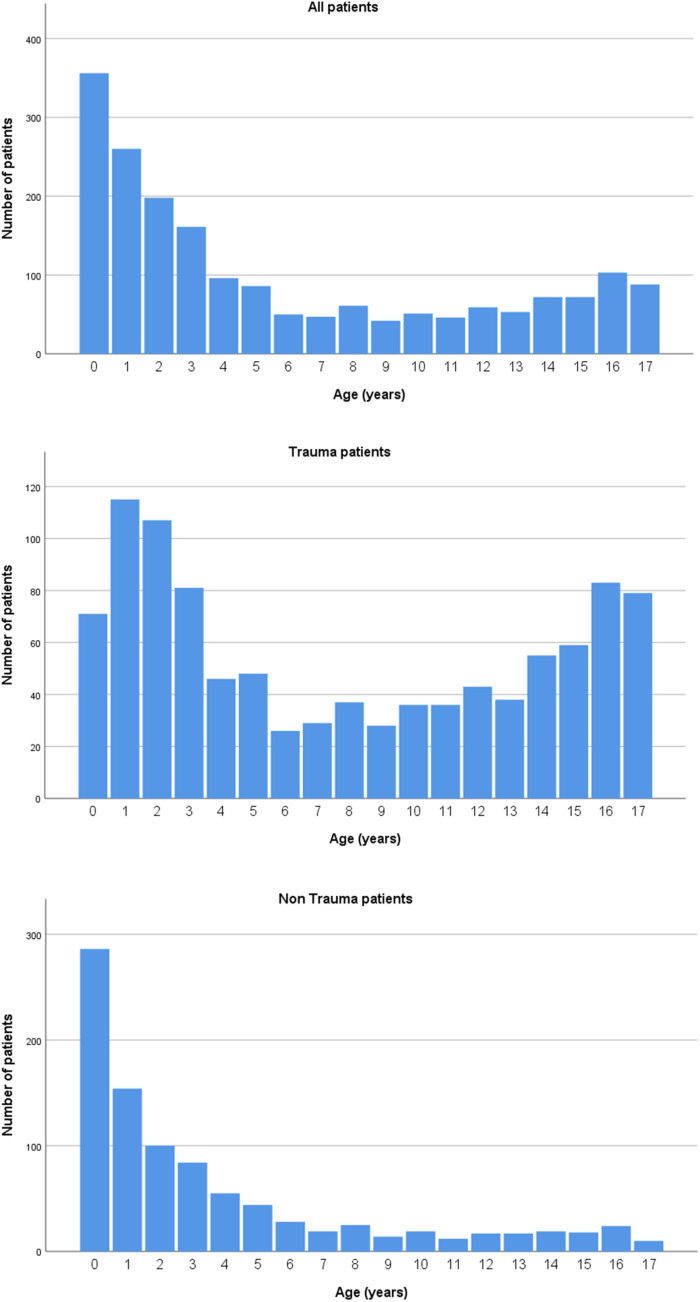
Age distribution in years (including estimated age)

**Fig. 2:**
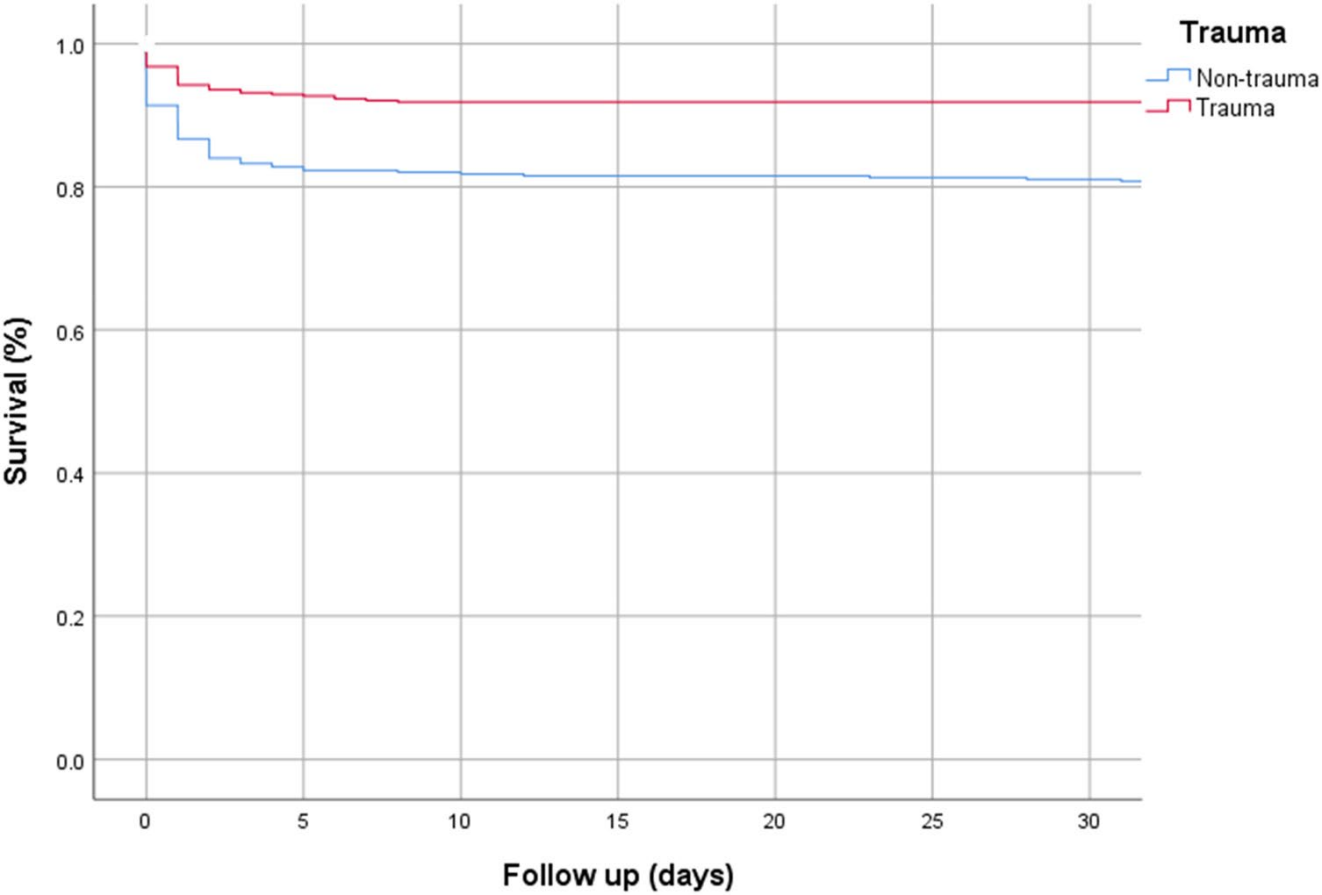
Kaplan–Meier survival

Statistics concerning patient and dispatch characteristics are described in Table 1. There were slightly more male patients with 59.1%. Most are ≤ 3 years of age, 53.2% (Fig. 2); with a median of 3.5 years. Average age was 5.8 years. The HEMS physician documented either the date of birth or the age in years; 4 patients were described as toddler or infant and, therefore, included without their precise age (Fig. 1).

**Table 1:** Patient and dispatch characteristics

Variable	*N* (%)
Gender (*n* = %)	
Male Female Unknown	1126 (59.1%) 770 (40.4%) 9 (0.5%)
Age (mean)	
Date of birth known (*n* = 1608) Including estimated age (*n* = 1901)	6.1 years 5.8 years
Medical history	
None Neurological Cardiac Pulmonary Syndrome Other Unknown	1046 (54.9%) 208 (10.9%) 47 (2.5%) 103 (5.4%) 67 (3.5%) 206 (10.8%) 337 (17.7%)
Medication use	
None Neurological Pulmonary Cardiac Other Unknown	1078 (56.6%) 100 (5.2%) 69 (3.6%) 15 (0.8%) 80 (4.2%) 586 (30.8%)
Dispatch reason	
Trauma Fall from height Traffic accident Burn wounds Drowning Violence Blunt trauma Stabbing Gunshot Sports Strangulation/hanging Inhalation trauma Explosion Other Non-trauma Sick child Resuscitation Respiratory insufficiency Lowered conscience Airway management Epilepsy Peri partum Anaphylaxis Intoxication Infections	1022 (53.6%) 404 (21.2%) 345 (18.1%) 91 (4.8%) 67 (3.5%) 34 (1.8%) 13 (0.7%) 15 (0.8%) 7 (0.4%) 31 (1.6%) 26 (1.4%) 5 (0.3%) 5 (0.3%) 48 (2.5%) 947 (49.7%) 461 (24.2%) 218 (11.4%) 188 (9.9%) 176 (9.2%) 147 (7.7%) 134 (7.0%) 52 (2.7%) 51 (2.7%) 18 (0.9%) 17 (0.9%)
Triage 2 patients 3 patients 4 patients 5 patients > 5 patients	136 (7.1%) 82 (4.3%) 22 (1.2) 13 (0.7%) 8 (0.4%) 11 (0.6%)

There is an equal distribution between trauma (53.5%) and non-trauma (49.7%) causes for the dispatch. For 62 patients (3.3%), there is an overlap between causes such as “resuscitation of a drowning patient”. Main reasons in the trauma group were; traffic accidents 345 (18.1%), falls from height 405 (21.2%) and burns in 91 patients (4.8%). In the group defined as non-trauma, main reasons were ‘sick’ children (24.2%), CPR (11.4%), and respiratory distress (9.9%).

Overall, 27.4% had known pre-existing medical conditions, most commonly a neurological condition (10.9%) or pulmonary diseases (5.4%).

For 136 dispatches (7.1%), more than one patient was attended by HEMS; not all other patients were children. For example, major road traffic accidents. In these dispatches, only patients requiring medical attention by HEMS were registered as patients.

### Prehospital interventions and medication

Prehospital interventions and administration of medication are described in Table 2. Most common route for administration of medication was intravenous access; this was performed in 862 (45.2%) patients. Intravenous access was placed in 758 patients (39.8%). Placement of intravenous access failed in 42 patients (2.2%); for 536 (28.1%) patients, no intravenous access was attempted. For 465 (24.4%), there were no recorded data about venous access or attempts. Intraosseous access was achieved in 175 (9.2%) patients for administration of medication.

**Table 2:** Prehospital interventions and medication (NA = not applicable according to EMS protocol)

	Parent/guardian	EMS	HEMS	Overall
IV access				
Overall Failed Unknown Intraosseous access Nasal medication				862 (45.2%) 42 (2.2%) 465 (24.4%) 175 (9.2%) 80 (4.2%)
Medication				
None	1833 (96.2%)	1569 (82.4%)	974 (51.1%)	799 (41.9%)
Paracetamol	8 (0.4%)	34 (1.8%)	99 (5.2%)	139 (7.3%)
Fentanyl		82 (4.3%)	415 (21.8%)	471 (24.7%)
Esketamine		15 (0.8%)	68 (3.6%)	83 (4.4%)
Lidocaine		*NA*	30 (1.6%)	30 (1.6%)
Benzodiazepines		126 (6.6%)	295 (15.5%)	397 (20.8%)
Anti-epileptics	53 (2.8%)	*NA*	2 (0.1%)	2 (0.1%)
Hypnotics		*NA*	230 (12.1%)	230 (12.1%)
Clonidine		*NA*	1 (0.1%)	1 (0.1%)
Dexmedetomidine		*NA*	2 (0.1%)	2 (0.1%)
Hyperosmolar therapy		*NA*	83 (4.4%)	83 (4.4%)
Muscle relaxant		*NA*	197 (10.3%)	197 (10.3%)
Epinephrine autoinjector	8 (0.4%)	*NA*	NA	8 (0.4%)
Adrenaline		60 (3.1%)	153 (8.0%)	199 (10.4%)
Atropine		1 (0.1%)	27 (1.4%)	28 (1.5%)
Amiodarone		2 (0.1%)	6 (0.3%)	8 (0.4%)
Noradrenaline		*NA*	13 (0.7%)	13 (0.7%)
Ephedrine		*NA*	23 (1.2%)	23 (1.2%)
Adenosine		*NA*	1 (0.1%)	1 (0.1%)
Vasopressin		*NA*	1 (0.1%)	1 (0.1%)
Dopamine		*NA*	3 (0.2%)	3 (0.2%)
Dobutamine		*NA*	11 (0.6%)	11 (0.6%)
Magnesium		*NA*	9 (0.5%)	9 (0.5%)
Calcium		*NA*	4 (0.2%)	4 (0.2%)
Bronchodilator (aerosol)	4 (0.2%)	22 (1.2%)	21 (1.1%)	41 (2.3%)
Budesonide		1 (0.1%)	3 (0.2%)	4 (0.2%)
Corticosteroids	2 (0.1%)	1 (0.1%)	25 (1.3%)	26 (1.4%)
Anti-histamine		9 (0.5%)	22 (1.2%)	32 (1.7%)
Tranexamic acid		2 (0.1%)	17 (0.9%)	19 (0.1%)
Anti-emetics		31 (1.6%)	130 (6.8%)	157 (8.2%)
Cyanokit		*NA*	1 (0.1%)	1 (0.1%)
Naloxone		*NA*	1 (0.1%)	1 (0.1%)
Flumazenil		*NA*	1 (0.1%)	1 (0.1%)
Thrombolysis		*NA*	2 (0.1%)	2 (0.2%)
Cephalosporin		*NA*	65 (3.4%)	65 (3.4%)
Entonox		1 (0.1%)	NA	1 90.1%)
Medication outside EMS protocol		*NA*	343 (18.0%)	343 (18.0%)
Airway procedures				
Laryngeal mask airway Intubation Cricothyrotomy		1 (0.1%) 64 (3.4%) *NA*	4 (0.2%) 295 (15.5%) 1 (0.1%)	5 (0.3%) 359 (18.8%) 1 (0.1%)
Surgical procedures				
Thoracotomy Thoracostomy Thoracic drain Thoracocenteses		*NA* *NA* *NA* 0 (0.0%)	1 (0.1%) 11 (0.6%) 5 (0.3%) 1 (0.1%)	1 (0.1%) 11 (0.6%) 5 (0.3%) 1 (0.1%)
Blood transfusion		*NA*	10 (0.5%)	10 (0.5%)

In 80 (4.2%) patients, nasal midazolam or fentanyl was administered.

Intubation was required for 359 (18.8%); 64 were intubated by EMS of which 35 intubations were supervised by HEMS physician. In five, EMS placed a laryngeal mask airway device; three of these patients were later intubated by HEMS. In one patient, intubation failed due to rigidity of the jaw and a cricothyrotomy was performed.

EMS crews mainly provided analgesia with paracetamol, fentanyl and S-Ketamine and bronchodilation with nebulizers or adrenaline inhalation (Table 2) according to EMS protocol and Advanced Life Support guidelines.

HEMS provided additional medications in the form of analgesia for many patients and adrenaline (Table 2). For 230 (12%) patients, additional sedation beyond EMS protocol was required with either etomidate or propofol; 197 (10.3%) received muscle relaxants such as rocuronium and suxamethonium. Two (0.1%) received thrombolysis for suspected pulmonary embolism based on transthoracic ultrasound made by the HEMS physician. A total of 343 (18.0%) of the patients received medication that is not available in the EMS protocol.

In 10 (0.5%) trauma patients, unmatched packed cells were administered on scene or during transport.

### Diagnosis

There is often an overlap in diagnosis. The most encountered problems were (advanced) airway management (*n* = 498, 26.1%), CPR (*n* = 245, 12.9%), traumatic brain injury of all severities (*n* = 375, 19.7%) and seizures (*n* = 358, 18.8%) (Table 3).

**Table 3:** Prehospital diagnosis and transport

Variable	*N* (%)
HEMS diagnoses prehospital	
Airway	
Compromised airway	498 (26.1%)
Breathing	
Pneumothorax Apnea Respiratory insufficiency Pneumonia Bronchospasm Hematothorax Fractured ribs Tension pneumothorax Inhalation trauma Tracheal malacia Laryngitis	30 (1.6%) 134 (7.0%) 89 (4.7%) 25 (1.3%) 15 (0.8%) 10 (0.5%) 7 (0.4%) 5 (0.3%) 1 (0.1%) 2 (0.1%) 1 (0.1%)
Circulation	
Cardiopulmonary resuscitation Shock Hypovolemic Infectious Obstructive Distributive Internal bleeding Cardiac problems (no resuscitation) External bleeding	245 (12.9%) 31 (1.6%) 18 (0.9%) 11 (0.6%) 3 (0.2%) 2 (0.1%) 39 (2%) 18 (0.9%) 7 (0.4%)
Disability	
Traumatic brain injury Seizures Confusion Skull fracture Intoxication Psychiatric Cerebral infection Spinal column fracture Subarachnoid bleeding (non trauma) Spinal cord injury Ischemic stroke Brain tumor complications	375 (19.7%) 358 (18.8%) 37 (1.9%) 30 (1.6%) 29 (1.5%) 22 (1.2%) 22 (1.2%) 18 (0.9%) 9 (0.5%) 8 (0.4%) 7 (0.4%) 6 (0.3%)
Other	
Infection Fracture (excl. spinal column fracture) Bruising Burn wounds Allergy Choking Asphyxia Hypothermia Transition after birth Apparent life-threatening event (ALTE) Hypoglycemia Hyperthermia Dehydration Crush injury Hyperventilation Luxation joint Traumatic amputation Diabetic ketoacidosis Migraine Mother peri-partum	156 (8.2%) 145 (7.6%) 112 (5.9%) 90 (4.7%) 60 (3.1%) 52 (2.7%) 35 (1.8%) 34 (1.8%) 29 (1.5%) 28 (1.5%) 15 (0.8%) 15 (0.8%) 10 (0.5%) 9 (0.5%) 8 (0.4%) 5 (0.3%) 5 (0.3%) 2 (0.1%) 1 (0.1%) 1 (0.1%)
Transport (*n* = 1905)	
EMS transport HEMS transport Ground transport Helicopter transport No transport indicated Deceased at the scene Unknown	811 (42.6%) 866 (45.5%) 820 (43.0%) 46 (2.4%) 98 (5.1%) 74 (3.9%) 56 (2.9%)

### Transport

After initial medical care by HEMS, 866 (45.5%) were transported accompanied by a HEMS physician.  This decision was based on the clinical judgement of the HEMS physician in 803 (42.2%) and requested by EMS 63 (3.3%). One patient was transported by HEMS in the rapid response vehicle due to lack of EMS availability. 74 (3.9%) patients died at the scene. All others were further cared for by EMS, 98 (5.1%) did not need (immediate) medical assistance and were not transported. For 56 patients (2.9%), it is unknown whether they were transported to a hospital. EMS transported 811 (42.6%) without HEMS assistance. For 46 (2.4%) patients, the HEMS crew decided to transport the patient by helicopter, mainly due to the distance to an appropriate hospital, such as a hospital with a pediatric intensive care unit (ICU) (Table 3).

### Hospital type, admittance, and ICU

Patients were transported to several hospitals in the Netherlands, 6 patients were transported to a hospital in Belgium due to the location of the incident (Table 4). The majority, 1043 (54.8%), were transported to a hospital with a pediatric ICU.

**Table 4:** Hospital type, admittance, ICU stay and mortality

Variable	*N* (%)
Hospital (*n* = 1905)	
Pediatric ICU available No pediatric ICU available Not transported to a hospital Deceased at the scene Neonatal ICU available (no pediatric ICU) Unknown	1043 (54.8%) 583 (30.6%) 160 (8.4%) 73 (3.8%) 1 (0.1%) 113 (5.9%)
Discharge from the ED (*n* = 884)	
Deceased in the ED Intensive care unit Intensive care unit other hospital High care unit Operating theater ICU after surgery Regular patient ward Patient ward other hospital Home	18 (2.0%) 324 (36.7%) 8 (0.9%) 11 (1.2%) 61 (6.9%) 38 (4.3%) 238 (26.9%) 86 (9.7%) 138 (15.6%)
Interventions in the ICU (*n* = 379)	
Duration of ICU stay (mean and range) Ventilation Duration (mean and range) ECMO Duration (mean and range)	6.2 days (0–123 days) 266 (14.0%) 4.3 days (0–70 days) 22 (5.6%) 2.8 days (0–7 days)
Mortality	
Overall (*n* = 1852 individual patients) Trauma (*n* = 1017) Non-trauma (*n* = 897)	176 (9.5%) 56 (5.5%) 137 (15.3%)

Of 884 (46.4%) emergency department (ED) charts were available, 861 (97.4%) initially presented at the Erasmus University Medical Center-Sophia Children’s Hospital, 23 (3.1%) other patients were transported to a hospital without pediatric ICU. From patients where ED charts were available 627 (71.0%), were transported with a HEMS physician. From the group of 884 patients, 324 (36.7%) were transferred to the ICU at the same hospital. Another 8 (0.9%) were initially transported to a hospital without an ICU or first to another hospital and later transferred to the Erasmus University Medical Center-Sophia Children’s Hospital. There were 61 (6.9%) patients who went directly to the operating theater from the emergency department; after surgery, another 38 (4.3%) of these patients were also admitted in the ICU.

In the ED, 18 (2.0%) of the patients died. From the ED, 238 (26.8%) were admitted to a regular ward; 138 (15.6%) were directly discharged home.

379 patients were admitted to the ICU, of whom some patients were not presented to the ED but directly transported to the ICU by HEMS. Ventilation was required in 266 (14.0%). Tracheal extubation was on the day of dispatch for 61 (32.3%) and day 1 post-dispatch for another 44 (23.3%). Extra-corporal membrane oxygenation (ECMO) was performed in 22 (5.6%). One child received a left ventricular assist device for severe cardiomyopathy before ECMO was discontinued.

### Mortality

Overall mortality was 176 (9.5%), from this group 99 (56.3%) died on the day of the dispatch, either at the scene, in the ED or in the ICU. Mortality was higher in the non-trauma group, 137 (15.3%), compared to 56 (5.5%) trauma patients. Considering that for some individual patients HEMS was deployed several times during the research period, we calculated mortality on individual patients instead of dispatches. The 1905 dispatched were for 1852 individual patients (Table 4). Of the 176 patients who died, 90.9% died in the first 7 days (Fig. 2).

## Discussion

This first large single region pediatric Dutch HEMS cohort shows that non-trauma patients account for nearly half of pediatric HEMS dispatches. Moreover, non-trauma mortality is much higher at 15.3 vs 5.5% for the trauma group. This difference is possibly caused by the severity of underlying illnesses in the non-trauma group and the high rate of CPR which has a poor prognosis [16, 17].

Our overall mortality of 9.3% is slightly higher than the 4.0–8.4% by HEMS in other countries [11–13]. This could be due to different selection criteria for HEMS dispatch or the high rate of non-trauma patients.

Fatality mainly occurred directly after dispatch (Fig. 2). This suggests that if the patient survived the initial days after trauma, prognosis in terms of survival is very good. In contrast, the non-trauma group showed a peak in mortality in the initial 7 days, but there was still a substantial group of patients who deceased later. Mainly attributed to preexistent causes.

Further studies should focus on finding preventive measures to reduce mortality for the non-trauma patient. These improvements could be focused on changes in dispatch criteria or additional training and information for all prehospital caretakers.

Although pediatric dispatches are 27% of the Rotterdam HEMS dispatches, continuous training remains essential considering the wide range of illnesses, injuries and age-specific characteristics. In each HEMS region, approximately 17,500 pediatric patients are attended to by EMS each year. This includes scheduled transports between health care facilities. EMS however rarely encounters a vitally compromised child, considering these numbers this gives an estimated < 2% of HEMS involvement in the total pediatric EMS calls [4, 6, 9, 10], of whom the majority are transferred back to EMS care. This shows that most patients can be handled by EMS within the national protocols.

Considering the high percentage of prehospital interventions and medications used outside of EMS protocol in this HEMS cohort, it remains essential for the dispatcher and EMS crews to be alert to signs of a seriously compromised child and to call in additional expertise from HEMS where necessary. In our view, this is an essential component of continuous training.

Standard procedure in the Netherlands for primary HEMS dispatch is aimed to prevent under-triage [7], resulting in unnecessary dispatches of HEMS. Considering the often incomplete information provided by the person making the emergency call, the high rate of 46.2% overall canceled dispatches when EMS is first to arrive at the scene is accepted in the Netherlands. 50.7% of whom care is transferred back to the care of EMS after assessment by HEMS.

Average age in this cohort at 6.1 years is comparable to previous studies [2, 9–12, 18, 19]. Remarkably patients of ≤ 3 years of age are responsible for 53.2% of the dispatches, possibly due to the dispatcher who has a lower threshold to dispatch HEMS in a sick or injured infant.

The airway was compromised in 515 (27%); this is due to a variety of reasons such as CPR, epilepsy, foreign body in the airway or lowered consciousness in trauma. Decision for prehospital intubation was made in 18.8% of patients by the HEMS physician. Compared to EMS studies with very low rate of airway procedures. This shows that this group of patients benefits from prehospital professionals who are experienced in solving airway problems in the pediatric population to prevent further deterioration [2, 4, 20].

Contrary to the high number of airway interventions, only 0.9% required prehospital surgical procedures, such as chest tubes or resuscitative thoracotomy. Previous Dutch HEMS study showed a low incidence of only 5 pediatric patients with a chest tube in 558 patients [18]. Illustrating that the individual HEMS physician does not encounter these procedures on regular basis in children. Depending on their experience with these procedures in adults this could be an area for additional training for the HEMS physician.

This cohort had a much higher CPR incidence of 12.9% compared to 1.9% in an Australian HEMS group [3]. Reasons for this could be different dispatch criteria and organization of prehospital care.

Prehospital medication is administered in 58.1% of patients. This number is higher compared to previous EMS studies [2, 10]. Medication in this HEMS cohort was either provided by parents/guardians, EMS and/or HEMS. We have no data concerning the medication provided by EMS in the general pediatric population.

Parents or guardians mainly provided benzodiazepines during seizures. EMS also mainly administered benzodiazepines to end seizures. Other frequently used medications were intravenous analgesia for pain relief and adrenaline in anaphylaxis or resuscitation. The Dutch EMS provides all medication according to their protocols per situation, and dosage is strictly limited based on body weight. HEMS provided intravenous analgesia in a much higher percentage of patients, for example, fentanyl was provided by EMS in 4.3% compared to 21.8% of patients by HEMS. In addition to medication that can be provided by EMS, HEMS also administered vasoactive medication, hypnotics and muscle relaxants for RSI, specific antidotes and antibiotics based on clinical judgement. In 18% of patients, medication that is not available for EMS, was administered.

After initial assessment 36.7% of patients were admitted to the ICU. Ventilation was required for 14% of the patients, this is slightly higher than the 12.8% that was intubated by HEMS. There were several patients intubated in the ED or later during the hospital stay in the ICU.

## Conclusions

This cohort shows that the ‘trauma-helicopter’ comprises more than injured children, considering half of the dispatches for non-trauma patients. We expect that ratio will be similar in other countries. This research shows that the non-trauma patient has a higher risk of mortality than the trauma patients. Further research is needed to determine why these patients are at higher risk of death. Possible reasons could be the focus on trauma patients by the dispatcher and first responders, with over-triage of the trauma patients or under-triage of the non-trauma patients. The non-trauma group is a heterogenic group who sometimes require very specific treatment based on medical history. Further study into the non-trauma group is needed to determine why these patients are at higher risk and improve training of EMS and HEMS personnel.

## Strengths and limitations

During the 6-year period of the study, we were able to include a very large group of 1905 pediatric dispatches. We were able to obtain in-hospital follow-up from the majority of patients transported by HEMS. A major limitation is the retrospective nature of this study. This could be responsible for a bias in, for example, documentation, because the HEMS physician usually only describes the major injuries and illnesses found in a patient. It is hereby possible that not all diagnosis and injuries are documented, especially in the patients who were quickly transferred to EMS care. Considering incomplete data concerning, for example, date of birth, we were unable to determine mortality of all patients. Furthermore, some patients did not have the Dutch nationality and we could not obtain follow-up all of these patients after hospital discharge or transfer to their home country.

## Abbreviations

HEMS: Helicopter Emergency Medical Services
EMS: Emergency Medical Services
CPR: Cardiopulmonary resuscitation
RSI: Rapid sequence intubation
ICU: Intensive Care Unit
ED: Emergency Department
ECMO: Extracorporal membrane oxygenation
